# Graph Theory-Based Brain Connectivity for Automatic Classification of Multiple Sclerosis Clinical Courses

**DOI:** 10.3389/fnins.2016.00478

**Published:** 2016-10-25

**Authors:** Gabriel Kocevar, Claudio Stamile, Salem Hannoun, François Cotton, Sandra Vukusic, Françoise Durand-Dubief, Dominique Sappey-Marinier

**Affiliations:** ^1^CREATIS Centre National de la Recherche Scientifique UMR5220 and Institut National de la Santé et de la Recherche Médicale U1206, INSA-Lyon, Université de Lyon, Université Claude Bernard-Lyon 1Lyon, France; ^2^Faculty of Medicine, Abu-Haidar Neuroscience Institute, American University of BeirutBeirut, Lebanon; ^3^Service de Radiologie, Centre Hospitalier Lyon-Sud, Hospices Civils de LyonLyon, France; ^4^Service de Neurologie A, Hôpital Neurologique, Hospices Civils de LyonLyon, France; ^5^CERMEP—Imagerie du Vivant, Université de LyonLyon, France

**Keywords:** MRI, multiple sclerosis, diffusion tensor imaging, structural connectivity, graph theory, classification, SVM

## Abstract

**Purpose:** In this work, we introduce a method to classify Multiple Sclerosis (MS) patients into four clinical profiles using structural connectivity information. For the first time, we try to solve this question in a fully automated way using a computer-based method. The main goal is to show how the combination of graph-derived metrics with machine learning techniques constitutes a powerful tool for a better characterization and classification of MS clinical profiles.

**Materials and Methods:** Sixty-four MS patients [12 Clinical Isolated Syndrome (CIS), 24 Relapsing Remitting (RR), 24 Secondary Progressive (SP), and 17 Primary Progressive (PP)] along with 26 healthy controls (HC) underwent MR examination. T1 and diffusion tensor imaging (DTI) were used to obtain structural connectivity matrices for each subject. Global graph metrics, such as density and modularity, were estimated and compared between subjects' groups. These metrics were further used to classify patients using tuned Support Vector Machine (SVM) combined with Radial Basic Function (RBF) kernel.

**Results:** When comparing MS patients to HC subjects, a greater assortativity, transitivity, and characteristic path length as well as a lower global efficiency were found. Using all graph metrics, the best *F*-Measures (91.8, 91.8, 75.6, and 70.6%) were obtained for binary (HC-CIS, CIS-RR, RR-PP) and multi-class (CIS-RR-SP) classification tasks, respectively. When using only one graph metric, the best *F*-Measures (83.6, 88.9, and 70.7%) were achieved for modularity with previous binary classification tasks.

**Conclusion:** Based on a simple DTI acquisition associated with structural brain connectivity analysis, this automatic method allowed an accurate classification of different MS patients' clinical profiles.

## Introduction

Multiple Sclerosis (MS) is a chronic disease of the central nervous system. It constitutes the leading cause of non-traumatic disability in young adults. While demyelination and inflammation are considered as initial and prominent mechanisms in relapsing-remitting (RR) MS, neurodegeneration is more present in progressive phases of MS, and probably constitutes the main cause of permanent disability accumulation (Mahad et al., 2015). Patients usually experience a first neurological episode known as a clinically isolated syndrome (CIS). This event evolves either into a relapsing-remitting course (85%) or a primary progressive (PP) course (15%). RR patients will evolve into a secondary progressive (SP) course after a period that could vary between 10 and 20 years.

MS diagnosis has been revolutionized in the last 30 years by the introduction of magnetic resonance imaging (MRI). *In vivo* detection of T2-weighted lesions and the assessment of their spatial and temporal distribution dominated the diagnostic criteria (Polman et al., 2015). However, the poor correlation of lesion load measurements with patients' disability remained an issue (Barkhof, 2002). The identification of this so-called “clinico-radiological paradox” has led to several studies utilizing a multitude of MRI strategies such as magnetization transfer, spectroscopy, and diffusion tensor imaging (DTI; Rovira et al., 2013). These techniques were successful in detecting alterations outside visible T2-lesions and contributed to our understanding of the pathological mechanisms occurring in normal appearing white matter (NAWM). To this end, DTI has been widely used to assess white matter damage in terms of myelin and axonal integrity. Both mean diffusivity (MD) and fractional anisotropy (FA) measurements have been shown to be mainly affected by myelin loss and/or decreased neuronal integrity (Hannoun et al., 2012). In addition, DTI offers the possibility to extract the trajectories of white matter pathways through the application of complex geometrical models (Tournier et al., 2012).

Based on the analysis of WM fibers networks, a simple description of structural brain connectivity was introduced through the application of a geometrical graph representation (Shuman et al., 2013). This graph theory approach has become a sensitive tool to detect alterations in brain pathologies by providing both local and global characterization of WM connections (Achard et al., 2012). Recently applied to MS patients, these methods demonstrated several alterations in brain connectivity (He et al., 2009; Richiardi et al., 2012; Li et al., 2013; Nigro et al., 2015; Romascano et al., 2015). Indeed, a negative correlation was reported between network efficiency and WM lesion load (He et al., 2009). Also, an increased local path length was highlighted in the hippocampus and the amygdala of MS patients with major depression (Nigro et al., 2015). Nonetheless, these few reports only focused on RR-MS patients.

In the present study, we propose to first characterize the structural connectivity in every clinical profile of MS patients by estimating global network metrics. Second, we describe a classification method to identify patient's clinical course using structural brain connectivity information. To our knowledge, this is the first attempt to solve this question in a fully automated manner. This attempt is based on the combination of graph-derived metrics with machine learning techniques using binary and multi-class classification tasks. Moreover, we introduce a non-empirical procedure to compute the best threshold for graph binarization.

In the first part of this paper, we describe our processing pipeline to generate graphs representing structural brain connectivity of each subject. Additionally, we provide a new approach to optimize the parameters in graph generation and binarization. In the second part, we describe several graph metrics to characterize brain network properties in the different MS clinical profiles. Finally, we describe our classification pipeline based on tuned support vector machine (SVM) with radial basic function (RBF) kernel.

## Materials and methods

### Subjects

Seventy-seven MS patients (24 RR, 24 SP, 17 SP, and 12 CIS; 29 men, 48 women; mean age 38.3 years, range 21.5–48.7) were recruited from the MS clinic of Lyon Neurological Hospital. Diagnosis and disease course were established according to the McDonald's criteria (Lublin and Reingold, 1996; McDonald et al., 2001). Disability was assessed with the Extended Disability Status Scale (median EDSS 4, range 0–7). Twenty-six healthy controls (HC) subjects, age and sex matched with the MS patients, were included in this study (9 men, 15 women; mean age 35.7 years, range 21.6–56.5). Demographics and clinical data are reported in Table 1 for each subjects' group. This prospective study was approved by the local ethics committee (CPP Sud-Est IV) and the French national agency for medicine and health products safety (ANSM). Written informed consent was obtained from all subjects.

**Table 1:** **Demographic information of MS patients of different clinical profiles (CIS, RR, SP, PP) and healthy controls (HC)**.

	***n***	**W/M**	**Age (years)**	**DD (years)**	**Median EDSS**
HC	24	15/9	35.7 ± 10.1	–	–
CIS	12	7/5	33.5 ± 6.4	2.8 ± 1.6	1.0 (0.0–3.0)
RR	24	20/4	35.1 ± 7.4	6.8 ± 4.1	2.5 (0.0–4.0)
SP	24	10/14	42.3 ± 4.4	13.8 ± 5.2	5.0 (4.0–7.0)
PP	17	11/6	40.9 ± 5.8	6.7 ± 3.2	4.0 (2.5–6.0)

Age and Disease Duration (DD) are expressed as mean ± standard deviation. Median Expanded Disability Status Scale (EDSS) along with its min and max values is also reported.

### MRI acquisition

MS patients and HC subjects underwent an MR examination on a 1.5T Siemens Sonata system (Siemens Medical Solution, Erlangen, Germany) using an 8-channel head-coil. The MR protocol consisted in the acquisition of a sagittal 3D-T1 sequence (1 × 1 × 1 mm^3^, TE/TR = 4/2000 ms) and an axial 2D-spin-echo DTI sequence (TE/TR = 86/6900 ms; 2 × 24 directions of gradient diffusion; *b* = 1000 s.mm^−2^, spatial resolution of 2.5 × 2.5 × 2.5 mm^3^) oriented in the AC-PC plane.

### Graphs generation

Cortical and sub-cortical gray matter (GM) segmentation was performed on 3D T1-weighted images using the Freesurfer image analysis suite (Reuter et al., 2012). The resulting segmentation was then used, first to define the graph nodes (*q* = *84*), and second, to classify voxels in four classes [white matter (WM), cortical GM, sub-cortical GM, cerebro-spinal fluid (CSF)] for tractography.

**Figure 1:**
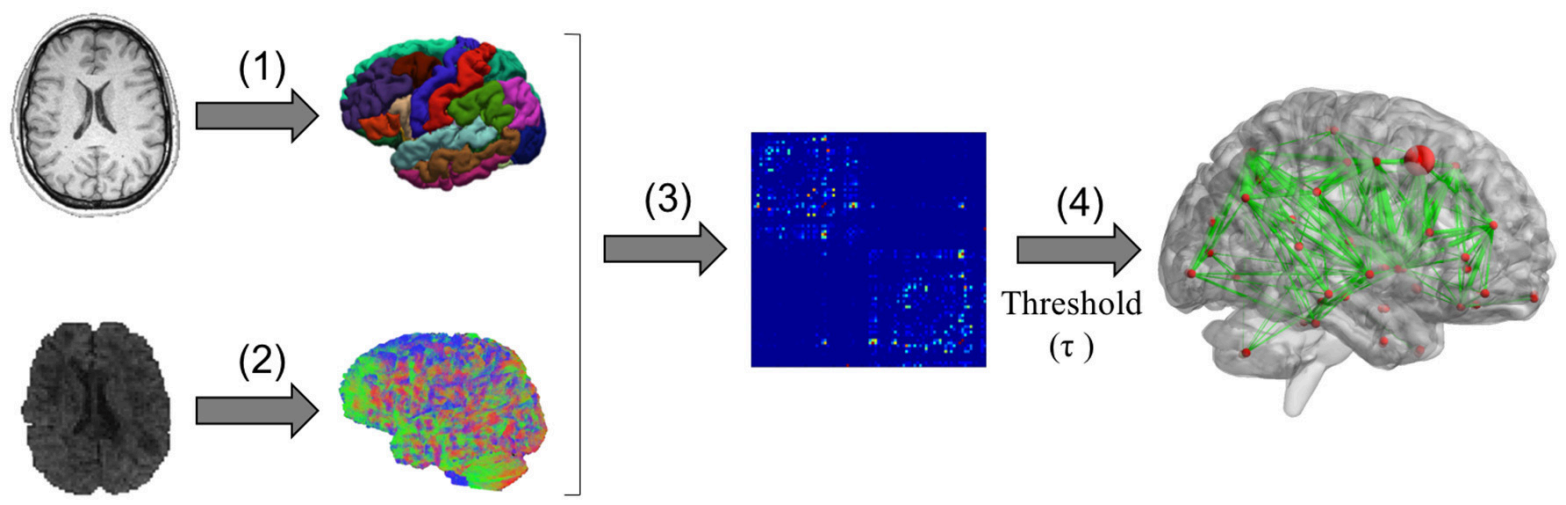
**Schematic representation of graph generation pipeline**. Graph nodes are generated through anatomical parcellation on T1 image (1) and probabilistic anatomically constrained streamline tractography is generated from diffusion images (2). Then, the numbers of streamlines connecting each pair of nodes are used to define edges in the weighted graph and generate the connectivity matrices. (3) Finally, a threshold τ is applied to the connectivity matrices to generate adjacency matrices (4).

Diffusion pre-processing steps consisted in Eddy current correction and non-brain voxels stripping using the FMRIB Software Library (FSL; Jenkinson et al., 2012). The main diffusion directions were then estimated in each voxel using diffusion orientation distribution function (dODF) computed with MRtrix (Tournier et al., 2012). Maximum spherical harmonics order *h*, defined as $\frac{\left(h+1\right)\left(h+2\right)}{2}<d$ with *d* the number of acquisition diffusion directions, was set to *h* = 4 to match with the acquisition protocol. Based on the four tissue-class segmentation, anatomically constrained probabilistic streamline tractography (ACT) was performed from dODF using the ACT package of MRtrix (Smith et al., 2012; Tournier et al., 2012). Connectivity matrices *A*∈ℕ^*q*×*q*^ were generated for each subject summing the number of streamline connecting each pair of nodes. Let *a*_*i, j*_1 ≤ *i, j* ≤ *q* be an element of *A*, then *a*_*i, j*_ = Ψ(*i, j*), where Ψ:ℕ^2^ → ℕ is the number of fibers connecting the node *i* with the node *j.* The connectivity matrix represent the adjacency matrix of the weighted undirected graph *G* = (*V, E*, ω) where *V* (|*V*| = *q*) is the node set containing the segmented GM brain regions, and *E* (|*E*| = *l*) is the graph edges set defined as:

$$\begin{array}{l} E=\left\{\left\{i,j\right\}\text{Ψ}\left(i,j\right)>0\forall \;1\leq i,j\leq q\right\} \end{array}$$

and ω:*E* → Ψ(*E*) is the weighted function that assigns at each edge *e*∈*E* its weight. Roughly speaking, this function is the same as Ψ but is defined only from the elements of the edges set *E.* In order to remove the weakest connections generated by the tractography, a threshold 0 ≤ τ ≤ 1 is applied on weighted graph *G*, generating an unweighted graph *G*′ = (*V*′, *E*′) (|*V*′| = *q*, |*E*′| = *l*′). As this threshold will affect network topology and density, it must be carefully chosen (Bullmore and Sporns, 2009; Simpson et al., 2013). From the two common methods used in brain network studies, namely absolute and proportional thresholding (Garrison et al., 2015), we choose to apply a proportional threshold function Ψ:*G*→*G*′ performing the following transformation:

$$ϒ:\{\begin{array}{c} V^{\prime }=V\\ E^{\prime }=L\left(\text{1},\ldots ,T\right),T=\frac{(q^{2}-q)\tau }{2} \end{array}$$

where *L* is the list of graph edges (*E*) sorted in ascending order of weight. In other words, this thresholding function only conserves the τ % of the strongest connections in the graph. For a better description a scheme of the graph generation pipeline is represented in Figure 1.

### Graph metrics estimation

Several graph metrics can be estimated to measure brain network properties (Rubinov and Sporns, 2010). In order to compute global graphs metrics, we first need to define three local properties of the graphs, including the degree *k*_*i*_ (number of connection) of the node *i*, the shortest path *d*_*ij*_ between the nodes *i* and *j* (Dijkstra, 1959), and finally the number of triangles^1^ *t*_*i*_ around the node *i*. In this work, the following six global graph metrics were computed:

**Graph density** *(D)* defined as the ratio between the numbers of effective connections in the graph to the number of possible connections.
  $$\begin{array}{l} D=\frac{l}{\frac{q^{2}-q}{2}} \end{array}$$
  **Assortativity** *(r)* (Newman, 2002) is the correlation coefficient between the degrees of two nodes at the extremities of an edge,
  $$r=\frac{\text{1/}l^{\prime }\sum_{\left(i,j\right)}kikj\;-\;[\text{1/}l^{\prime }\sum_{\left(i,j\right)\in E^{\prime }}\frac{1}{2}(k_{i}\text{+}k_{j}){]}^{2}}{\text{1/}l^{\prime }\sum_{\left(i,j\right)}\frac{1}{2}(k_{i}^{2}\text{+}k_{j}^{2})\;-\;[\text{1/}l^{\prime }{\sum_{\left(i,j\right)\in E^{\prime }}\frac{1}{2}(k_{i}\text{+}k_{j})]}^{2}}$$
**Transitivity** *(T*) is the ratio between the number of triangles and the number of triplets in the graph,
  $$T=\frac{\sum_{i\in V^{\prime }}2t_{i}}{\sum_{i\in V^{\prime }}k_{i}(k_{i}\;-\text{ 1})}$$
**Global efficiency** (*E*)_*g*_ is the mean of the inverse of the shortest paths in the graph,
  $$E_{g}=\frac{1}{q}\sum_{i\in V^{\prime }}E_{i}=\frac{1}{q}\sum_{i\in V^{\prime }}\frac{\sum_{j\in V^{\prime },j\neq i}d_{ij}^{-\text{1}}}{n-\text{1}}$$
**Modularity** *(Q)* is the capability of a network to be separated into modules.
  $$Q=\sum_{u\in M}\left[e_{uu}\;-{\left(\sum_{v\in M}e_{uv}\right)}^{2}\right]$$
  where *M* represent the number of non-overlapping modules in the network, and *e*_*uv*_ the proportion of links connecting nodes *u* and *v* inside the module.
**Characteristic path length (CPL)** is the mean of the shortest paths in the graph
  $$CPL=\frac{1}{q}\sum_{i\in V^{\prime }}L_{i}=\frac{1}{q}\sum_{i\in V^{\prime }}\frac{\sum_{j\in V^{\prime },j\neq i}d_{ij}}{q-\text{1}}$$
  where *L*_*i*_ is the average distance between node *i* and all other nodes.

All the metrics, except graph density, were computed based on the binarized graph *G*′ using the brain connectivity toolbox on MATLAB (Rubinov and Sporns, 2010).

### Classification using support vector machine (SVM)

Support Vector Machines (SVM) is a family of supervised classification algorithms (Cortes and Vapnik, 1995). The idea behind SVM classifier is to find the best hyperplane to separate data belonging to two different classes. More in detail, let *S* = {(*x*_1_, *y*_1_), …, (*x*_*n*_, *y*_*n*_)} be a set of instances where $x_{i}\in {\mathbb{R}}^{m}$ and $y_{i}\in {\left\{-1,1\right\}}^{m}$, a “soft margin” SVM classifier is based on the solution of the following optimization problem:

$$\begin{array}{c} {\text{min}}_{w,b}\frac{1}{2}{\left\|w\right\|}^{2}+C\sum_{i=1}^{n}\epsilon_{i}\\ subject\;to\;y_{i}\left(w^{T}\phi \left(x_{i}\right)+b\right)\geq 1-\;\epsilon_{i},i\;=1,\ldots ,n\\ \epsilon_{i}\geq \text{0 }i\;=1,\ldots ,n \end{array}$$

Where ϵ is a relaxation variable of the optimization problem and *C* is the error penalization constant. The function ϕ(*x*_*i*_) is a mapping function that maps the feature vector *x*_*i*_ to a higher dimensional space.

The Lagrangian duality formulation of this problem is:

$$\begin{array}{c} {\text{max}}_{\alpha }\sum_{i=1}^{n}\alpha_{i}\;-\;\frac{1}{2}\sum_{i,j=1}^{n}y_{i}y_{j}\alpha_{i}\alpha_{j}\langle \phi \left(x_{i}\right),\phi \left(x_{j}\right)\rangle \\ subject\;to\;0\leq \alpha_{i}\leq C,i=1,\ldots ,n\\ \sum_{i}^{n}\alpha_{i}y_{i}\;=0 \end{array}$$

where α_*i*_ are Lagrange multipliers. We can rewrite the inner product 〈ϕ(*x*_*i*_), ϕ(*x*_*j*_)〉 as a function *K*(*x, y*) = (ϕ(*x*)^*T*^ϕ(*y*)) called kernel.

Different kernel functions, mapping input space in higher dimensional space, are described in literature: polynomial, RBF, sigmoid functional, and others (Gärtner, 2003). In this work, we used RBF kernel defined as:

$$K\left(x,y\right)=\text{exp}\left(-{\left\|x-y\right\|}^{2}\text{ * }\gamma \right)$$

RBF kernel was selected due to its good performances. Indeed, the analysis reported in Keerthi and Lin (2003), indicates that if complete parameters selection using the Gaussian kernel has been conducted, there is no need to consider linear SVM. Moreover, since the number of feature is not large, mapping to a higher dimensional space helps to improve the performances, as reported in Hsu et al. (2008). In order to find the optimal input parameters of SVM, namely *C* and γ, grid search was performed using growing sequences of *C* and γ. More in detail, we used the range [2^−5^, 2^15^] for *C* and [2^−15^, 2^3^] for γ. Generalization of classification performances was ensured by *K*-Fold cross validation using 10-folds. Finally, each feature was standardized in order to improve the quality of the classification.

Performance measurements used in this work are based on the analysis of true positive (TP), true negative (TN), false positive (FP), and false negative (FN) instances classified during the testing phase. Precision, recall, and *F*-Measure were used to measure the classification performances. More in details, precision reflects the fraction of retrieved instances that are correctly classified, and it is defined as $\frac{\text{TP}}{\text{TP}+\text{FP}}.$ Recall represents the portion of positive instances that are correctly identified and it is defined as $\frac{\text{TP}}{\text{TP}+\text{FN}}$. Finally, *F*-Measure was obtained combining precision and recall and is defined as $2*\frac{\left(\text{precision}*\text{recall}\right)}{\left(\text{precision}+\text{recall}\right)}$.

### Statistical analysis

In order to detect if graph metrics are suitable to differentiate MS clinical profiles, classic statistical analyses were performed using R (R Developement Core Team, 2015). Dependence of global graph metrics with patient's age and gender was assessed, by fitting a general linear model (GLM) to the data and by estimating the relative importance of clinical course age and gender in these models. Normality of the residues was checked for each fit. Wilcoxon-Mann-Whitney test was conducted to test the differences between the global metrics measured between the subject's groups. The tests were computed with a level of significance of 5%. Numerical results are presented as mean ± *SD*.

## Results

### Number of fibers and threshold definition

In graph generation, the definition of two parameters (*f*, the number of fibers and τ, the threshold value of binarization) is crucial since no consensus has been reported yet in the literature. To this end, variability studies were performed in order to measure these two parameters' effects on graph metrics.

**Figure 2:**
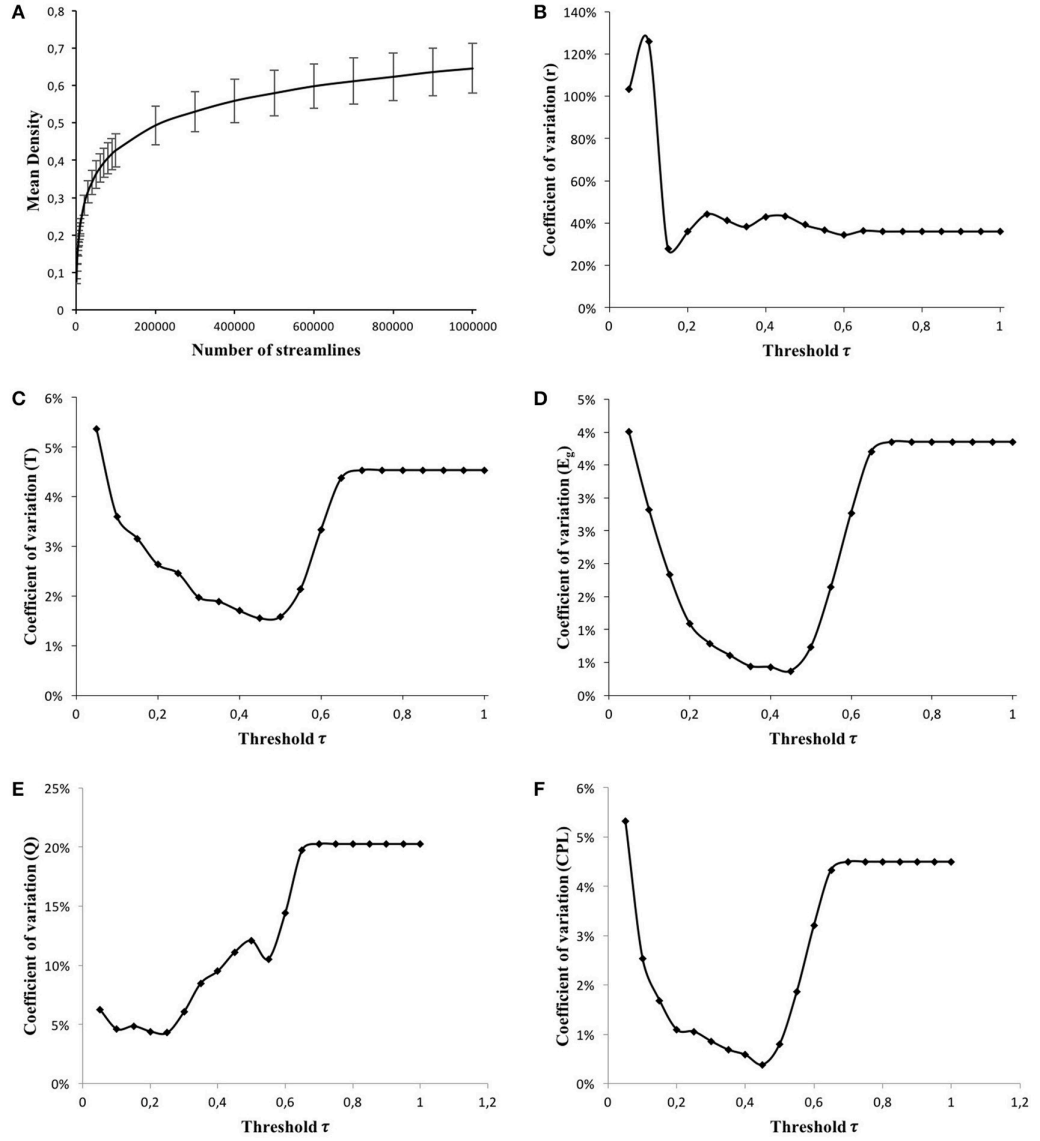
**Evolution of the mean density, estimated on weighted graphs, respect to the number of generated streamlines *f* (A)**. Evolution of the coefficients of variations of the global networks metrics [Assortativity **(B)**, Transitivity **(C)**, Global Efficiency **(D)**, Modularity **(E)**, and Characteristic Path Length **(F)**] respect to the applied threshold τ.

First, the optimal number of streamlines generated by tractography was chosen in a range in which the variability of graphs' density is stable. In order to compute the optimal range, several graphs were generated on HC data using different numbers of fibers (*f*) (from 1000 to 10,000 every 1000, from 10,000 to 100,000 every 10,000, and from 100,000 to 1,000,000 every 100,000). Figure 2A represents the evolution of the mean graph densities, along with their respective standard deviations, with respect to the number of fibers *f*. From 1000 to 90,000 fibers, the graph density varies from 0.08 to 0.43. In contrast, for higher number of fibers (100,000 to 1,000,000), the graph density becomes stable. Thus, we chose to generate 500,000 fibers per patient, corresponding to a mean density of 0.58 for HC graphs.

Second, the optimal threshold value τ applied to weighted graphs was chosen following two criteria: (1) the inter-subject variability of all the estimated global metrics is as low as possible and (2) the metric variability is stable over the threshold range. Coefficients of variation (CV) of five global graph metrics (assortativity, efficiency, transitivity, modularity, and characteristic path length) were estimated on HC graphs for different threshold values (from τ = 0.05 to τ = 1 with a 0.05 step). As shown in Figures 2B–F, the highest CVs were found for the smallest values of τ (from 0.05 to 0.2). τ values varying from 0.65 to 1 were rejected since they are higher than graph density (0.58). This behavior is also confirmed by the plots in Figures 2B–F where the CV is stabilized for a threshold τ > 0.65, and does not modify the topology of the graph. Thus, we chose to apply a threshold τ of 0.35 (35%) corresponding to a mean CV of 0.90% on HC graphs.

### Global graph metrics

**Figure 3:**
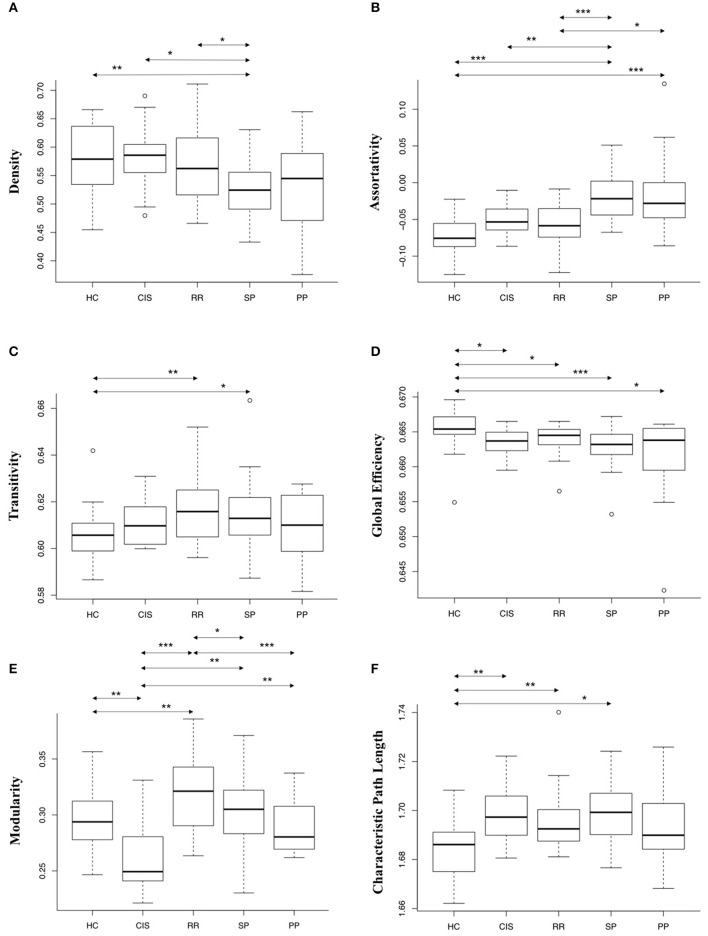
**Box-plots of the global network metrics [Density (A), Assortativity (B), Transitivity (C), Global Efficiency (D), Modularity (E), and Characteristic Path Length (F)] estimated on unweighted graphs (except for graph density)**. Differences between the different clinical groups were tested using a Wilcoxon Mann-Whitney test (*p < 0.05; **p < 0.01; ***p < 0.001).

As shown in Figure 3, significant differences were found in several graph metrics when comparing MS and HC groups. As reported in Tables 2, 3, density was decreased in SP patients (*p* < 0.01). Assortativity was increased in SP (*p* < 0.001) and PP patients (*p* < 0.001). Transitivity was increased in RR (*p* < 0.01) and SP patients (*p* < 0.05). Global efficiency was decreased in CIS (*p* < 0.05), RR (*p* < 0.05), SP (*p* < 0.001), and PP patients (*p* < 0.05). Characteristic path length was increased in CIS (*p* < 0.01), RR (*p* < 0.01), and SP patients (*p* < 0.05). Finally, modularity was decreased in CIS patients (*p* < 0.01) and increased in RR patients (*p* < 0.01).

**Table 2:** **Mean (±*SD*) values of global metrics [density (D), assortativity (r), transitivity (T), efficiency (E_*g*_), Modularity (Q), and characteristic path length (CPL)] were calculated on unweighted graphs (except for graph density)**.

	***D***	***r***	***T***	***E_g_***	***Q***	**CPL**
HC	0.58 ± 0.06	−0.07±0.028	0.61 ± 0.01	0.67 ± 0.00	0.29 ± 0.02	1.68 ± 0.01
CIS	0.58 ± 0.06	−0.05±0.025	0.61 ± 0.01	0.66 ± 0.00	0.26 ± 0.03	1.70 ± 0.01
RR	0.56 ± 0.06	−0.06±0.028	0.62 ± 0.01	0.66 ± 0.00	0.32 ± 0.03	1.69 ± 0.01
SP	0.52 ± 0.05	−0.02±0.034	0.61 ± 0.01	0.66 ± 0.00	0.30 ± 0.03	1.70 ± 0.01
PP	0.53 ± 0.08	0.02 ± 0.058	0.61 ± 0.01	0.66 ± 0.01	0.29 ± 0.02	1.69 ± 0.02

**Table 3:** **Statistical significances (*p*-values) when comparing the global graph metrics [density (D), assortativity (r), transitivity (T), efficiency (E_*g*_), Modularity (Q), and characteristic path length (CPL)] between different clinical profiles of MS patients and Healthy Controls (HC)**.

***p*-value**	***D***	***r***	***T***	***Eg***	***Q***	**CPL**
HC-CIS	–	–	–	0.01279	0.007523	0.003987
HC-RR	–	–	0.005906	0.01406	0.003158	0.003519
HC-SP	0.002123	0.0000001043	0.01188	0.0003185	–	0.0001363
HC-PP	–	0.000511	–	0.01023	–	–
CIS-RR	–	–	–	–	0.00008845	–
CIS-SP	0.02294	0.005575	–	–	0.003997	–
CIS-PP	–	–	–	–	0.009331	–
RR-SP	0.02269	0.00008197	–	–	0.03941	–
RR-PP	–	0.01546	–	–	0.0007993	–
SP-PP	–	–	–	–	–	–

Significant differences were also found when comparing global graph metrics in between MS patients' groups. Density was lower in SP group compared to CIS and RR groups (*p* < 0.05). Assortativity was higher in SP group compared to CIS (*p* < 0.01) and RR (*p* < 0.001) groups, and in PP compared to RR groups (*p* < 0.05). Modularity was lower in CIS group compared to RR (*p* < 0.001), SP (*p* < 0.01), and PP groups (*p* < 0.01), and higher in RR group compared to SP (*p* < 0.05) and PP groups (*p* < 0.001). No significant changes were identified between PP and SP groups.

Effects of disease course, age, and gender on graph metrics were estimated using General linear models. The relative importance of these predictors was estimated for each graph metric, as reported in Table 4. A mean value of 86.26, 9.55, and 4.19% was obtained for the disease course, patient age, and gender predictors, respectively. These results showed that global graph metrics are not significantly dependent on age and gender. Moreover, head patient movements were estimated from the transformation matrices generated during the Eddy current correction. For each subject, Root Mean Square deviation was computed. Mean displacement values (mean ± *SD*) were the following for each group: HC 1.32 ± 0.51 mm; CIS: 2.04 ± 0.72 mm; RR: 1.91 ± 0.93 mm; SP: 2.04 ± 0.69 mm; PP: 1.80 ± 0.57 mm. No difference was found when comparing head displacements between subjects' groups.

**Table 4:** **Relative importance (%) of the clinical course, age, and gender predictors in the general linear models with as response, density (D), assortativity (r), transitivity (T), global efficiency (E), modularity (Q), and characteristic path length (L)**.

**Predictors**	***D***	***r***	***T***	***E***	***Q***	***L***	**Mean**
Course	71.05	79.18	93.91	92.69	95.41	85.34	86.26
Age	9.76	19.07	5.47	6.40	2.96	13.63	9.55
Gender	19.19	1.75	0.61	0.91	1.63	1.04	4.19

### SVM classification

The SVM classifier described in the Materials and Methods Section was applied on graph metrics. Different binary classification (HC-CIS, CIS-RR, RR-PP, RR-SP, and SP-PP) and a multi-class classification (CIS-RR-SP) tasks were performed. Each classification task was tested using either only one metric or all the metrics at the same time. Results on classification tasks are reported in Table 5.

**Table 5:** **Precision, Recall, and *F*-Measure (%) for classification tasks with different global metrics [density (D), assortativity (r), efficiency (Eg), transitivity (T), modularity (Q), and characteristic path length (CPL)] and with all the six global metrics at the same time (All)**.

		***D***	***r***	***T***	***E_g_***	***Q***	**CPL**	**All**
HC—CIS	Precision	44.0	80.2	67.3	64.3	83.9	76.1	**92.0**
	Recall	66.7	80.6	69.4	66.7	83.3	75.0	**91.7**
	*F*-Measure	53.0	80.4	68.3	65.5	83.6	75.5	**91.8**
CIS—RR	Precision	53.6	72.9	67.3	67.3	89.0	55.6	**92.0**
	Recall	55.6	72.2	69.4	69.4	88.9	61.1	**91.7**
	*F*-Measure	54.6	72.5	68.3	68.3	88.9	58.2	**91.8**
RR—PP	Precision	67.5	73.0	40.8	66.1	70.7	63.2	**75.6**
	Recall	65.9	68.3	51.2	65.9	70.7	63.4	**75.6**
	*F*-Measure	66.7	70.6	45.4	66.0	70.7	63.3	**75.6**
RR—SP	Precision	66.8	**85.5**	41.6	54.3	68.8	62.9	68.8
	Recall	66.7	**85.4**	41.7	54.2	68.8	62.5	68.8
	*F*-Measure	66.7	**85.4**	41.6	54.2	68.8	62.7	68.8
SP—PP	Precision	54.0	**67.5**	65.4	56.2	65.4	55.5	59.6
	Recall	56.1	**65.9**	65.9	58.5	65.9	58.5	61.0
	*F*-Measure	55.0	**66.7**	65.6	57.3	65.6	57.0	60.3
	Precision	42.5	61.7	38.3	38.9	55.9	42.7	**71.3**
CIS-RR-SP	Recall	53.3	61.7	38.3	48.3	55.0	53.3	**70.0**
	*F*-Measure	47.3	61.7	38.3	43.1	55.4	47.4	**70.6**

Best performances in bold.

Highest classification performances were achieved using all the graph metrics as feature vector. The following classification tasks: HC-CIS, CIS-RR, RR-PP, and CIS-RR-SP, obtained the best *F*-Measures of 91.8, 91.8, 75.6, and 70.6%, respectively. Using only one graph metric, the best performances of the previous binary classification tasks were achieved for the modularity metric, reaching *F*-Measures of 83.6, 88.9, and 70.7%, respectively. For the multi-class classification task, the best performances were achieved for the assortativity metric, reaching a *F*-Measure of 61.7%.

For the classification tasks RR-SP and SP-PP, the highest classification performances were achieved for the modularity metric, with *F*-Measures of 85.4 and 66.7%, respectively. For this two tasks, low but still acceptable classification performances were reached using all the graph metrics as feature vector, with *F*-Measures of 68.8 and 60.3% respectively.

## Discussion

Graph theory analysis offers new opportunities to identify potential biomarkers for the characterization of global as well as local effects of pathological mechanisms on brain networks. This is particularly of great interest in MS patients, who are subject to inflammatory and demyelinating processes leading to detectable local WM lesions, but also to global microscopic neurodegenerative damage. In this work, we used global graph-derived markers first, to characterize the four standard MS clinical profile and second, to automatically classify MS patients accordingly. A previous study attempted to characterize graph metrics changes in MS patients, and furthermore classify patients using these metrics (Muthuraman et al., 2016). While this study reached good classification performances, it was limited by the consideration of only two clinical profiles of MS patients (CIS and RR MS patients). To our knowledge, the present study is the first to characterize and classify patients from all the MS clinical courses.

In this work, we described a fully automated pipeline to generate structural connectivity graphs from T1-weighted and diffusion images. Two major parameters for tractography (number of fibers *f*) and graph generation (binarization threshold τ), were optimized and set as follows: *f* = 500,000 and τ = 0.35. Six global graph metrics were then computed and analyzed to characterize tissue damages in the four MS clinical profile. The resulting graph metrics were used to automatically classify MS patients using SVM combined with RBF kernel. Again, an accurate parameter selection was performed using grid search on the two SVM parameters (*C* and γ).

First, we showed significant differences in graph metrics between MS clinical profiles and HC, and to highlight the sensitivity of this approach to characterize pathological alterations. Indeed, global efficiency was decreased while transitivity, assortativity, and characteristic path length were increased in brain networks of MS patients compared to HC subjects. These changes in the measure of node integration (decreased global efficiency and increased characteristic path length; Achard and Bullmore, 2007), observed in all MS patients, may correspond to transient damages caused by inflammatory and demyelinating processes. This result is in agreement with a previous study (Shu et al., 2011), reporting decreased global and local efficiencies correlated with EDSS, patients disease duration, and WM lesion load. Moreover, transitivity, a measure of network redundancy, was increased in RR patients, reflecting the ability of RR patients to compensate for transitory myelin damages and recruit new pathways. This observation tends to disappear in SP patients who showed in contrast a density decrease, probably due to the accumulation of successive attacks and Wallerian degeneration. Assortativity was increased in progressive courses, SP and PP, compared to HC. This result may reflect the neurodegenerative mechanisms leading to the segregation of two sub-networks, composed either by high or low degree nodes. One should notice that assortativity in HC networks is negative, reflecting the existence of connections between nodes with different degrees, as previously reported as a characteristic of biological networks (Newman, 2002). Modularity, which reflects the integration and segregation levels between graph sub-networks, was significantly higher in RR patients compared to HC. This increase is probably related to active focal inflammatory mechanisms, as shown by the presence of gadolinium-enhancing lesions. In contrast, modularity was significantly lower in CIS patients compared to HC. This decrease is probably related to early diffuse inflammation. These findings lead to the hypothesis that an increased modularity is a consequence of focal disconnections due to WM lesions, while a decreased modularity is resulting from a global and diffuse inflammation. If this result is further confirmed, it means that modularity could represent a sensitive and specific marker of the initial inflammatory phase occurring in CIS patients. However, the small number of CIS patients does not allow us to further emphases this finding.

Second, we showed the high capability of SVM methods in combination with graph-derived metrics, to automatically classify MS patients according to their clinical profile. The high levels of accuracy and *F*-Measure confirmed the potential of our method to accurately classify MS patients. Moreover, the good classification results, obtained using all graph-derived metrics as feature vectors, showed the complementarity of these metrics to better characterize MS pathological alterations and better differentiate MS clinical profile.

In this study, we focalized our classification analysis on the following tasks: HC-CIS, CIS-RR, RR-PP, RR-SP, SP-PP, and CIS-RR-SP, due to their strong clinical interest. First, the main challenge of the neurologist is to differentiate CIS patients from HC subjects. Second, CIS-RR and RR-SP classification tasks were performed to test the capability of our method to differentiate two “successive” clinical courses of the disease. These classification tasks are of great clinical interest, as the clinician needs to identify a patient conversion from CIS to RR, and from RR to SP in order to start or adapt the medical treatment. While CIS-RR classification task reached excellent performances (*F*-Measure of 91.8%), probably due to the few WM alterations usually observed in CIS patients compared to RR patients, the RR-SP task only reached modest, but still reasonable, performances (*F*-Measure of 68.8% with all graph metrics). The difficulty to differentiate RR to SP patients may be a consequence of the comparable WM alterations in those two clinical profiles. Indeed, the difference between RR and SP patients mainly resides in the progression and the accumulation of disability caused by WM damages in the progressive form. A longitudinal study may help to take this aspect into account and increase classification performances. Third, the differentiation of SP-PP was realized to test the sensitivity of this method to discriminate two progressive profiles, as proposed by the new MS phenotype classification (Lublin et al., 2014). This concept may explain the relatively weak performances obtained during the classification task (*F*-measure of 60.3% with all graph metrics). In the future, we plan to distinguish progressive patients by the presence or not of inflammation. Moreover, we plan to increase our sensitivity by classifying SP and PP courses based on MS longitudinal data. Finally, the classification of RR-PP was tested to evaluate our method's capability to separate the two starting MS profile, sharing a similar mean disease duration (6.8 years for RR and 6.7 years for PP). Furthermore, we performed a multi-class classification, CIS-RR-SP, between three successive clinical profiles. Considering the difficulty to obtain good performances with multi-class classification, this *F*-Measure (70.6%) constitutes a relative satisfactory result, which confirms the capability of this approach to discriminate MS clinical courses.

### Methodological limitations

From the methodological perspective, this study may suffer from several limitations. The first one consists in the arbitrary choice of parcellation method for graph nodes definition. Indeed, organizational network parameters are influenced by the spatial scale (number of nodes) of the network (Zalesky et al., 2010). However, this study showed that comparisons can be performed between graphs of same spatial scale, which is the case in the present study. Second, the small number of patients could cause biases such as over fitting. However, we minimized these potential biases by carefully selecting the parameters of the SVM classifier and using *K*-Fold cross-validation to generalize classification results. Further, the small number of each patient profile may not reflect the general population and induce biases in graph metrics results. Third, the diffusion acquisition was performed using 24 directions, which is relatively low compared to current standards in diffusion imaging. Hence, we tried to minimize crossing-fibers error by using spherical deconvolution instead of classical diffusion tensor model. In future, we plan to improve the robustness of the proposed method by enlarging the sample size of patients with a new dataset acquired using high angular resolution diffusion imaging (HARDI) at 3T. It should be noted that tractography can be severely modified by the lesions occurring in MS patients WM. Indeed, WM damages (MRI detectable lesion and MRI undetectable damage in NAWM) lead to changes in diffusion. These alterations induce modifications in the dODF and thus in the reconstructed streamlines. As consequence, the graphs measurements obtained from tractography are modified by WM lesions as well as by MRI undetectable alterations. In a future study, in order to measure the effects of such MRI undetectable tissue alterations we plan to mask WM lesions. Moreover, we performed only a binary class and a multi-class classification tasks in certain sub-groups without classifying all the groups together. This choice was first led by clinical interests. Indeed, only certain clinical profiles need to be classified, such as successive clinical courses of the disease (i.e., CIS-RR-SP) or differential diagnosis (i.e., RR-PP). Second, from a methodological point of view, the patients' sample was too small for a five multi-class classification task.

## Conclusion

We proposed a graph-based method, to first, characterize brain WM damages in MS patients using graph metrics of structural brain connectivity, providing a great potential for MS patient discrimination. Second, the use of “data-driven” methods such as machine learning algorithm is suitable for the classification of complex diseases like MS. As a major improvement, this approach allowed to obtain excellent *F*-Measures that are probably due to the high sensitivity of the six structural graph metrics. However, these preliminary findings would need to be confirmed on large database of MS patients such as the French OFSEP project (Cotton et al., 2015). Therefore, these findings demonstrated that the high performance of SVM classification methods combined with the high sensitivity of global graph metrics provided a sensitive and automated tool to classify MS patients' clinical profiles.

## Author contributions

CS, GK developed the method and performed the classification, the statistical analysis, and wrote the paper. SH helped to write the paper. FD provided the data and helped in the interpretation of the results. FC, SV, and DS helped to write the paper and provided the clinical expertise in the interpretation of the results.

### Conflict of interest statement

The authors declare that the research was conducted in the absence of any commercial or financial relationships that could be construed as a potential conflict of interest.

## Footnotes

^1^In graph theory a triangle is a sub-graph composed by three vertices and three edges.
